# Development and internal validation of risk prediction model of metabolic syndrome in oil workers

**DOI:** 10.1186/s12889-020-09921-w

**Published:** 2020-11-30

**Authors:** Jie Wang, Chao Li, Jing Li, Sheng Qin, Chunlei Liu, Jiaojiao Wang, Zhe Chen, Jianhui Wu, Guoli Wang

**Affiliations:** 1 School of Public Health, North China University of Science and Technology, No.21 Bohai Avenue, Caofeidian New Town, Tangshan City, Hebei Province 063210 P.R. China; 2 Hebei Province Key Laboratory of Occupational Health and Safety for Coal Industry, North China University of Science and Technology, Tangshan, Hebei P.R. China; 3 College of Science, North China University of Science and Technology, Tangshan, Hebei P.R. China

**Keywords:** Data mining, Oil workers, Metabolic syndrome, Risk prediction

## Abstract

**Background:**

The prevalence of metabolic syndrome continues to rise sharply worldwide, seriously threatening people’s health. The optimal model can be used to identify people at high risk of metabolic syndrome as early as possible, to predict their risk, and to persuade them to change their adverse lifestyle so as to slow down and reduce the incidence of metabolic syndrome.

**Methods:**

Design existing circumstances research. A total of 1468 workers from an oil company who participated in occupational health physical examination from April 2017 to October 2018 were included in this study. We established the Logistic regression model, the random forest model and the convolutional neural network model, and compared the prediction performance of the models according to the F1 score, sensitivity, accuracy and other indicators of the three models.

**Results:**

The results showed that the accuracy of the three models was 82.49,95.98 and 92.03%, the sensitivity was 87.94,95.52 and 90.59%, the specificity was 74.54, 96.65 and 94.14%, the F1 score was 0.86,0.97 and 0.93, and the area under ROC curve was 0.88,0.96 and 0.92, respectively. The Brier score of the three models was 0.15, 0.08 and 0.12, Observed-expected ratio was 0.83, 0.97 and 1.13, and the Integrated Calibration Index was 0.075,0.073 and 0.074, respectively, and explained how the random forest model was used for individual disease risk score.

**Conclusions:**

The study showed that the prediction performance of random forest model is better than other models, and the model has higher application value, which can better predict the risk of metabolic syndrome in oil workers, and provide corresponding theoretical basis for the health management of oil workers.

**Supplementary Information:**

The online version contains supplementary material available at 10.1186/s12889-020-09921-w.

## Background

Metabolic syndrome (MetS) refers to the accumulation of multiple metabolic risk factors in the body including obesity, impaired glucose regulation, dyslipidemia and hypertension. MetS is a group of complex clinical syndromes based on insulin resistance. Relevant literatures have shown that metabolic syndrome increases the risk of cardiovascular disease, type 2 diabetes and chronic kidney disease [1–3].With the social and economic development and changes in people’s lifestyles, the prevalence of metabolic syndrome has increased year by year and brought a heavy economic burden, which has become an important health issue of common concern to people worldwide.

At present, the definition and diagnostic criteria of metabolic syndrome have not been completely unified. In 1998, WHO officially named the “metabolic syndrome” and and proposed corresponding diagnostic criteria for the first time [4].Over the course of the next decade, the diagnostic criteria for metabolic syndrome have undergone many changes and revisions, including 2001 national cholesterol education program adult treatment group report for the third time (NCEP ATP III). Chinese diabetes association (CDS) diagnostic criteria in 2004. International diabetes federation (IDF) diagnostic criteria 2005. In 2009, the American heart association (AHA), the international diabetes federation, the national heart, lung and blood institute and other institutions jointly proposed a tentative unified standard [5–8].According to a large number of epidemiological data, the global prevalence of MetS is about 30 %[9]. DoosupShin based on 2007–2014 national health and nutrition survey data on MetS prevalence statistics found that American adults MetS prevalence rate has reached 34.3% (according to the revised NCEP-ATP III diagnostic criteria) [10].In South Korea, according to the same diagnostic criteria, the prevalence rate of metabolic syndrome in adults from 2009 to 2013 was as high as 30.52 %[11].In China, in 2010, Jieli L u[12] and others conducted a data report analysis of 97,098 adults in China, and estimated the prevalence of MetS was 33.9% (according to the NCEP-ATP III diagnostic criteria).In 2015, Ting Liu analyzed the prevalence of MetS among 34,025 residents in Jilin province and found that the prevalence of MetS was 32.5% (according to IDF diagnostic criteria) [13].In 2016, Ri L i[14]and others conducted a meta-analysis showing that the prevalence of MetS in subjects over 15 years old was 24.5% (according to IDF diagnostic criteria).Although the diagnostic criteria are not uniform, it is undeniable that metabolic syndrome has become one of the chronic diseases with high incidence in China and even in the world.

Data mining refers to extracting hidden information and knowledge with potential research value from large data, which is often used in the medical field with large amounts of data and fast update speed. Among them, the classification algorithm has been widely concerned and applied in recent years. This algorithm takes a variety of risk factors affecting the occurrence of disease as a prerequisite, and uses statistical methods and computer algorithms to build a predictive model of disease risk. The constructed model is used to predict the probability of a certain population or individual developing a certain disease, and then provides a theoretical basis for personal health management and corresponding preventive measure s[15].At present, Logistic regression, Cox regression, BP neural network, decision tree, support vector machine and other models are mostly used to construct metabolic syndrome risk models at home and abroad [16–18]. These models can be used to identify high-risk groups of MetS, persuade them to change their unhealthy lifestyles, reduce and slow down the occurrence and development of the disease. Among the m[19–21], Logistic regression and Cox regression, as traditional statistical modeling methods, are widely used and have strong explanatory power. However, Cox regression is often used for survival analysis data, which requires two dependent variables at the same time and has relatively strict requirements on data. The decision-making tree model has strong visibility, but is prone to overfitting and poor generalization effect. The random forest model is a classifier composed of multiple decision-making trees, which improves the weak generalization ability of a single decision-making tree and balances the error of unbalanced data. As a kind of artificial neural network model, BP neural network is fault-tolerant to some extent, but local minimization problems often occur, and the learning speed is slow, and the phenomenon of overfitting is easy to occur. In the convolutional neural network model, the local receptive field and weight sharing of convolutional kernel reduce the computational complexity and have high accuracy and good generalization ability. Due to regional and cultural differences, the effects of existing models vary, and mature and accurate metabolic syndrome risk prediction systems have not been established at home and abroad. Moreover, most of these models established at present are aimed at the assessment of the risk of disease in the general population, ignoring the special group of occupational population.

As an important part of China’s non-renewable energy industry, the petroleum industry still accounts for a large proportion in the national economy. Oil workers are also the main laborers in the production of the secondary industry in China. Their health will affect the development of China’s economy to a certain extent and should be paid more attention. Oil workers are affected by high temperature, noise, shift work and other harmful occupational factors, as well as a variety of adverse lifestyles caused by occupational stress, which can greatly increase the incidence of metabolic syndrome to some extent. For special occupational group, the risk prediction model of ordinary people is no longer suitable for them, so it is necessary to establish a risk prediction model of metabolic syndrome for them, so as to achieve early detection, diagnosis and treatment, and protect the health of oil workers. In this study, a certain oil industry worker was selected as the research object, and the traditional Logistic regression model, random forest model and the recent thermal convolutional neural network model were developed and internally verified. The prediction performance of each model is compared to find the optimal model, which provides a theoretical basis for the health management of this special occupation group of oil workers.

## Methods

### Data sources and research objects

This study strictly followed the Transparent Reporting of a Multivariable Prediction Model for Individual Prognosis or Diagnosis (TRIPOD) reporting guidelines and prepared this report according to the relevant guideline s[22]. This study adopted the existing circumstances research method. In order to avoid the contingency caused by manual partition of the data set and make the sample more representative, this study adopted 10-fold cross validation (The data set was divided into 10 mutually exclusive subsets on average. The union of 9 subsets was taken as the training set each time, and the remaining 1 subset was the test set.The cycle was repeated 10 times to represent the final performance of the model by the average of all test results) for data partition and internal verification, so as to further improve the robustness of model prediction.

A total of 1468 workers from an oil company who attended occupational examination and physical examination from April 2017 to October 2018 were selected as the research objects. Inclusion criteria: length of service 1 year or above. Aged between 18 and 60. Complete questionnaire and physical examination data. All subjects gave their informed consent for inclusion before they participated in the study. The study was conducted in accordance with the Declaration of Helsinki, and the protocol was approved by the Ethics Committee of North China University of Science and Technology(NO.16040).

### Outcomes and predictor variables

One-to-one questionnaire survey was conducted on oil workers by uniformly trained personnel to collect the following information: ① General situation: gender, age, education, income status, marital status, etc. ② Lifestyle: smoking, drinking, diet and physical exercise. ③ history of personal and family diseases: hyperglycemia, hypertension, hyperlipidemia, etc. ④Working conditions: shifts, exposure to high temperature, noise and other harmful factors. ⑤Physical examination: height, weight, blood pressure and waist circumference, etc.

The study subjects took venous blood in the early morning after fasting for 12 h, and tested the biochemical indicators such as fasting blood glucose, high-density lipoprotein, and triglyceride using the Dirion CS-1200 automatic biochemical analyzer (China Changchun Dirion Medical Technology Company). The diagnostic criteria of metabolic syndrome [8] can be diagnosed if it meets three or more of the following five indicators:

I. Central obesity: Chinese people have a waist circumference ≥ 85 cm (male). waist circumference ≥ 80 cm (female).

II. Elevated blood glucose: FBG ≥5.6 mmol/L or those who have been diagnosed with diabetes and receive treatment.

III. TG ≥ 1.7 mmol/L or those who have been diagnosed with hypertriglyceridemia and received treatment.

IV. HDL-C < 1.04 mmol / L (male). HDL-C < 1.30 mmol/L (female) or those who have been diagnosed with low-density lipoproteinemia and received treatment.

V. Systolic / diastolic blood pressure ≥ 130/85 mmHg or those diagnosed with hypertension and receiving treatment.

### Quality control

The investigators can only take up their posts after unified training. The collected questionnaire data are collected on the spot for double and double check and input, and the questionnaires with incorrect input are checked for the third time to ensure the accuracy of the collected data. The same instrument was used for physical examination and laboratory test, and the biochemical indicators were tested by the same kit in North China Petroleum Underground Hospital.

### Sample size

Through consulting a large number of relevant literatures, it was found that there were about 15 predictive factors related to metabolic syndrome.General neural network and random forest model require that the sample content is more than 2 times of explanatory variables.The newly developed Logistic regression model *R*^2^_CS_adj_ (the estimated measure after adjusting the overfitting of the model) is at least 0.1, so to achieve the expected contraction coefficient of 0. 9 [23], we finally need a sample size of at least 1274.

### Statistical methods

CscrMainUI system developed by a scientific research company was used to scan and input questionnaires and establish a database. IBM SPSS19.0 was used for statistical analysis. The measurement data obeying the normal distribution were expressed as $\overline{\mathrm{x}}$ ±s, and the t test was used for comparison between groups. The non-normally distributed measurement data were represented by [M (P25,P75)], and the rank sum test was used for comparison between groups. The count data were used as the ratio, and Pearson x^2^ test was used for comparison between groups. Unconditional binary classification logistic regression was used for multivariate analysis. The independent variable introduction criterion was *P* ≤ 0.05, and the test level α = 0.05(both sides).

### Establishment and validation of the models

Input variables of the three models: predictors of metabolic syndrome of oil workers determined by multivariate logistic regression analysis and results of a large number of relevant literature reviews. The output variable was whether metabolic syndrome occurred.

First of all, logistic regression model, random forest model and convolutional neural network model were respectively constructed by Python’s Numpy. Use regularization techniques for the three models respectively (that is, by increasing the training error and reducing the test error to constrain the parameters to be optimized) to prevent over-fitting. After that, the data set was divided into 10 parts, one part as test set and the other nine parts as training set. The three training models were created by using the functions of Python Sklearn library to train the training set, and the test data were used for model evaluation and prediction. Finally, the average of ten operations was taken as the true index of the model. During the establishment of logistic regression model, the corresponding function of Sklearn library was called to establish the training model. For the construction of random forest, in order to ensure the robustness of the model, the Shuffle function in sklearn library was used to disrupt the data set. In order to ensure the randomness of data selection, the Gini coefficient was used as the classification index to predict the model. In the construction of the convolutional neural network model, the input characteristics were first standardized, ${x}^{\ast }=\frac{x-\min }{\max -\min }$, the original data was normalized to the interval [0,1], and two pooling layers and two full connection layers were used. The size of the output activation was calculated according to the size of the input activation (W), the size of the receiving domain of the convolutional layer neurons (F), the step size they apply (S), and the size of the zero fill used on the boundary (P). The formula was (W ‐ F + 2P)/S + 1. The relu function f(*x*) = max(0, *x*) was used as the excitation function to further reduce the error, and the maximum pooling was used in the pooling process. Finally, the matplotlib library was used to visualize the three models respectively and calculate the confusion matrix of the results. The internal validation of the model was carried out by 10-fold cross-validation, and the sensitivity, specificity, F1 score, area under ROC curve, Brier score, observed-expected ratio and other indicators of the three models were compared.

## Results

### General situation

**Fig. 1:**
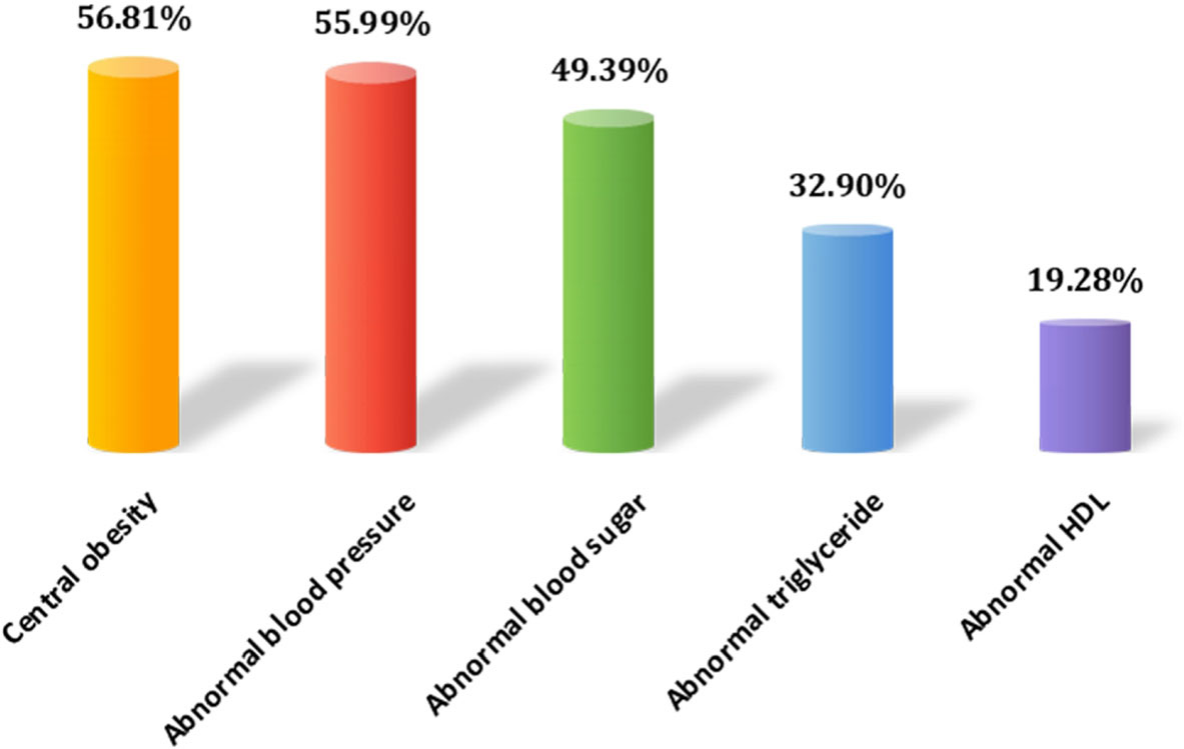
Comparison of abnormal rates among components of metabolic syndrome

Of the 1468 oil workers, 1105 were male, with an average age of 43(38,48),363 were women, with an average age of 44(42,47). The prevalence rate of metabolic syndrome in petroleum workers was 40.67%, among which, the rate of central obesity was 56.81%, the rate of abnormal blood glucose was 49.39%, the rate of abnormal triglyceride was 32.90%, the rate of abnormal HDL was 19.28%, and the rate of abnormal blood pressure was 55.99%. As shown in Fig.1.

### Independent variable screening

Single factor analyses were performed on the basic conditions, diet and lifestyle, occupational exposure factors and laboratory tests of 1468 oil workers. The results showed statistically significant differences in age, gender, Body Mass Index(BMI), marital status, family history of hypertension, family history of diabetes mellitus, salt, meat intake, smoking status, drinking status, shift work situation, Occupational heat, noise, hemoglobin, uric acid(UA), alanine transaminase(ALT), etc. (*P* < 0.05), are shown in Tables 1, 2, 3 and 4.

**Table 1:** Comparison of the basic conditions of oil workers with and without metabolic syndrome

Basic conditions	Category(Unit)	MetS n(%)/M(P_25_,P_75_)		*χ*^*2*^*/Z*	*P*
		No	Yes		
Age	Year	43(38,47)	44(40,49)	−5.79	< 0.001
Gender	Male	601(69.00)	504(84.42)	45.26	< 0.001
	Female	270(31.00)	93(15.58)		
BMI	Kg/m^2^	23.9(21.90,25.90)	26.80(24.90,28.80)	−16.35	< 0.001
Marital status	Unmarried	56(6.43)	15(2.51)	11.82	0.003
	Married	782(89.78)	559(93.63)		
	Others	33(3.79)	23(3.85)		
Education level	Junior high school and below	133(15.27)	104(17.42)	9.07	0.011
	High school/technical secondary school	374(42.94)	290(48.58)		
	College and above	364(41.79)	203(34.00)		
Per capita monthly household income(Yuan)	< 2000	619(71.07)	454(76.05)	8.05	0.018
	2000~	212(24.34)	109(18.26)		
	3000~	40(4.59)	34(5.70)		
Family history of hypertension	No	489(56.14)	288(48.24)	8.88	0.003
	Yes	382(43.86)	309(51.76)		
Family history of hyperlipidemia	No	801(91.96)	538(90.12)	1.51	0.22
	Yes	70(8.04)	59(9.88)		
Family history of diabetes mellitus	No	725(83.24)	454(76.05)	11.58	0.001
	Yes	146(16.76)	143(23.95)

**Table 2:** Comparison of diet and lifestyle of oil workers with and without metabolic syndrome

Factors	Category	MetS n(%)/M(P_25_,P_75_)		*χ*^*2*^	*P*
		No	Yes		
Salt	Light	221(25.37)	88(14.74)	26.39	< 0.001
	Moderate	381(43.74)	276(46.23)		
	Salty	269(30.88)	233(39.03)		
Meat intake	Never	23(2.64)	13(2.18)	9.38	0.025
	Occasionally	198(22.73)	101(16.92)		
	Regularly	335(38.46)	232(38.86)		
	Every day	315(36.17)	251(42.04)		
Fruit intake	Never	37(4.25)	27(4.52)	6.59	0.086
	Occasionally	278(31.92)	223(37.35)		
	Regularly	258(29.62)	146(24.46)		
	Every day	298(34.21)	201(33.67)		
Dairy intake	Never	127(14.58)	103(17.25)	119.81	< 0.001
	Occasionally	230(26.41)	297(49.75)		
	Regularly	199(22.85)	111(18.59)		
	Every day	315(36.17)	86(14.41)		
Carbonated beverage intake	Never	370(42.48)	270(45.23)	10.52	0.015
	Occasionally	384(44.09)	258(43.22)		
	Regularly	79(9.07)	31(5.19)		
	Every day	38(4.36)	38(6.37)		
Physical exercise	No	307(35.25)	259(43.38)	9.90	0.002
	Yes	564(64.75)	338(56.62)		
Smoking status	No smoking	524(60.16)	262(43.89)	39.30	< 0.001
	Quit smoking	51(5.86)	61(10.22)		
	Smoking	296(33.98)	274(45.90)		
Drinking status	No drinking	585(67.16)	309(51.76)	37.02	< 0.001
	Alcohol withdrawal	16(1.84)	24(4.02)		
	Drinking	270(31.00)	264(44.22)

**Table 3:** Comparison of occupational exposure factors of oil workers with and without metabolic syndrome

Factors	Category	MetS n(%)/M(P_25_,P_75_)		*χ*^*2*^	*P*
		No	Yes		
Shift work situation	Never	535(61.42)	254(42.55)	51.44	< 0.001
	Once	208(23.88)	202(33.84)		
	Now	128(14.70)	141(23.62)		
Labour intensity	Mild	93(10.68)	44(7.37)	5.36	0.069
	Moderate	434(49.83)	295(49.41)		
	Severe	344(39.49)	258(43.22)		
Occupational heat	No	548(62.92)	266(44.56)	48.34	< 0.001
	Yes	323(37.08)	331(55.44)		
Noise	No	429(49.25)	206(34.51)	31.39	< 0.001
	Yes	442(50.75)	391(65.49)

**Table 4:** Comparison of laboratory tests in oil workers with and without metabolic syndrome

Biochemical Indicators	MetS n(%)/M(P_25_,P_75_)		*Z*	*P*
	No	Yes		
RBC(× 10^12^/L)	5.01(4.65,5.33)	5.29(4.99,5.54)	−6.94	< 0.001
MCV(fl)	88.80(85.10,92.00)	88.20(84.80,91.80)	−0.85	0.397
BPC(×10^12^/L)	256.00(219.50,290.75)	251.00(211.00,284.00)	−0.55	0.59
MPV(fl)	8.20(7.70,8.80)	8.20(7.70,8.80)	−0.83	0.405
Hemoglobin(g/L)	155(141,165)	160(151,169)	−6.44	< 0.001
TBIL(mmol/L)	13.50(10.50,17.70)	13.45(10.30,17.10)	−0.81	0.421
UA(mmol/L)	307(242,373)	367(304,426)	−11.13	< 0.001
ALT(U/L)	20.00(14.00,24.00)	35.00(21.00,45.00)	−17.07	< 0.001

The significant factors of univariate analysis were included in the multivariate nonconditional Logistic regression analysis. The results showed that the risk of metabolic syndrome increased with age, BMI, UA and ALT. People with a family history of diabetes, a strong salt taste, occasional consumption of dairy products, daily consumption of carbonated beverages, smoking, shift work, and exposure to high temperatures are more likely to develop metabolic syndrome. The protective factors of metabolic syndrome include family income of 2000–3000 yuan per capita, daily consumption of dairy products and physical exercise. Combined with the results of relevant literature review, 13 significant factors in the multivariate analysis were taken as independent variables for the establishment of the model, as shown in Tables 5 and 6.

**Table 5:** Multivariate nonconditional Logistic regression analysis of influencing factors in oil workers with metabolic syndrome

Factors	*B*	*S.E*	*Waldχ*^*2*^	*P*	*OR*	*95%CI*
Age	0.088	0.012	55.251	0.000	1.092	1.067, 1.118
Per capita monthly household income(2000~)	−0.77	0.22	12.244	0.000	0.463	0.301, 0.713
Per capita monthly household income(3000~)	0.166	0.388	0.184	0.668	1.181	0.552, 2.525
BMI	0.273	0.026	114.091	0.000	1.313	1.249, 1.381
Family history of diabetes mellitus	0.373	0.183	4.129	0.042	1.452	1.013, 2.080
Salt(Moderate)	0.86	0.206	17.429	0.000	2.362	1.578, 3.536
Salt(Salty)	0.555	0.214	6.759	0.009	1.742	1.146, 2.648
Dairy intake(Occasionally)	0.676	0.216	9.771	0.002	1.966	1.287, 3.003
Dairy intake(Every day)	−1.149	0.261	19.317	0.000	0.317	0.190, 0.529
Carbonated beverage intake(Every day)	1.102	0.365	9.148	0.002	3.012	1.474, 6.153
Physical exercise	−0.398	0.152	6.86	0.009	0.672	0.499, 0.905
Smoking status(Smoking)	0.431	0.181	5.675	0.017	1.539	1.079, 2.194
Shift work situation(Once)	0.974	0.172	32.184	0.000	2.648	1.892, 3.707
Shift work situation(Now)	1.509	0.237	40.489	0.000	4.522	2.841, 7.198
Occupational heat	0.656	0.224	8.548	0.003	1.926	1.241, 2.989
UA	0.004	0.001	27.244	0.000	1.004	1.003, 1.006
ALT	0.029	0.005	40.946	0.000	1.030	1.020, 1.039

**Table 6:** Assignment of influencing factor variables

Variable name	Variable meaning	Assignment method
Y	MetS	0 = No,1 = Yes
X_1_	Age	Continuous variable (year)
X_2_	Per capita monthly household income	1 = < 2000,2 = 2000–3000,3 = ≥3000
X_3_	BMI	Continuous variable(Kg/m2)
X_4_	Family history of diabetes mellitus	1 = No,2 = Yes
X_5_	Salt	1 = Light,2 = Moderate,3 = Salty
X_6_	Dairy intake	1 = Never,2 = Occasionally,3 = Regularly,4 = Every day
X_7_	Carbonated beverage intake	1 = Never,2 = Occasionally,3 = Regularly,4 = Every day
X_8_	Physical exercise	1 = No,2 = Yes
X_9_	Smoking status	1 = No smoking,2 = Quit smoking,3 = Smoking
X_10_	Shift work situation	1 = Never,2 = Once,3 = Now
X_11_	Occupational heat	1 = No,2 = Yes
X_12_	UA	Continuous variable(mmol/L)
X_13_	ALT	Continuous variable(U/L)

### Collinearity diagnosis

The diagnosis of collinearity was made by using the binary correlation coefficient r, tolerance and variance inflation factor(VIF).The results showed that the correlation coefficient |r| was 0.31 at most and |r| < 0.5, as shown in Supplementary Table 1, Additional File 1. The minimum tolerance was 0.844, much higher than 0.1, and the maximum variance inflation factor was 1.185, less than 5, as shown in Supplementary Table 2, Additional File 2. The above results indicate that there is no serious multicollinearity among the screened independent variables.

### Evaluation of model results

In this study, the sample data were divided by the 10-fold cross-validation method. Three models, namely Logistic Regression, Random Forest and CNN, were established respectively to learn and predict the data set. The prediction results of each model were compared with the actual results of the sample, so as to obtain the respective confusion matrix of the model, as shown in Table 7. Among the three models, age, ALT, BMI and UA all rank in the top four in terms of the importance of predictive variables, as shown in Supplementary Figure 1–3, Additional Files 3, 4 and 5.

**Table 7:** Sample classification results of Logistic regression model, Random Forest model and Convolutional neural network model [N (%)]

Model	predictive value	Actual value		
		Yes	No	Total
Logistic regression model	Yes	766(87.94)	152(25.46)	918
	No	105(12.06)	445(74.54)	550
	Total	871	597	1468
Random forest model	Yes	832(95.52)	20(3.35)	852
	No	39(4.48)	577(96.65)	616
	Total	871	597	1468
CNN	Yes	789(90.59)	35(5.86)	824
	No	82(9.41)	562(94.14)	644
	Total	871	597	1468

**Fig. 2:**
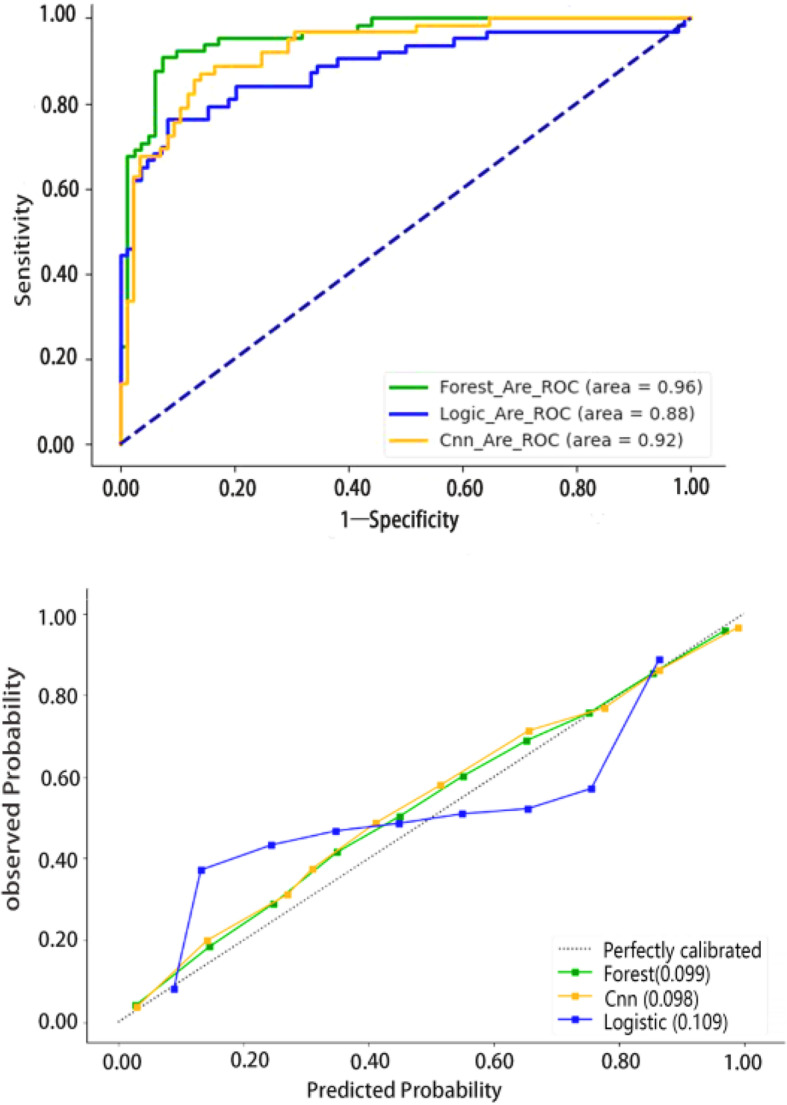
ROC curves and calibration curves of three predictive models

The accuracy of three models, Logistic Regression Model, Random Forest Model and CNN, was 82.49, 95.98 and 92.03%, respectively. The sensitivity was 87.94, 95.52 and 90.59%, respectively. The specificity was 74.54, 96.65 and 93.10%, respectively. F1 Score was 0.86, 0.97 and 0.93 respectively. The area under ROC curve was 0.88, 0.96 and 0.92, respectively. The Brier score of the three models was 0.15, 0.08, 0.12, observed-expected ratio was 0.83, 0.97, 1.13, real-in-the large was 0.109, 0.099, 0.098, ICI was 0.075, 0.073, 0.074, respectively. The calibration diagrams of logistic regression model, random forest model and CNN model were all close to the diagonal, and there was no serious deviation from the calibration results. The random forest model performs better than Logistic Regression model and CNN in both discrimination and calibration. See Table 8 and Fig.2.

**Table 8:** Comparison of predictive performance of the three models

Evaluation index	Logistic regression model	Random forest model	CNN
Accuracy rate(%)	82.49	95.98	92.03
Sensitivity(%)	87.94	95.52	90.59
Specificity(%)	74.54	96.65	94.14
F1 Score	0.86	0.97	0.93
AUC	0.88	0.96	0.92
Brier score	0.15	0.08	0.12
observed-expected ratio	0.83	0.97	1.13
calibration-in-the-large	0.109	0.099	0.098
Integrated Calibration Index	0.075	0.073	0.074

### Random forest model for individual risk score calculation

The random forest risk prediction model established in this study was the optimal model suitable for risk prediction of metabolic syndrome in oil workers. To further apply the model to reality and calculate the individual risk score, predict_proba method of the Sklearn library in Python can be used. The dependent variable Y is whether the person has metabolic syndrome, and the independent variable X is the 13 predictive factors in this study, and then the risk score of a certain person or a group of people can be obtained. The higher the score, the greater the risk of the disease. As shown in Table 6, Code IV, Additional File 6.

## Discussion

At present, all countries in the world have recognized that the establishment of disease risk prediction model has a greater role in preventing and controlling the occurrence of metabolic syndrome, and established the corresponding MetS model based on the epidemiological data. In 2008, Fabien Szabo DE Edelenyi et al. in France conducted a large case-control study and found that the prediction accuracy of metabolic syndrome status using random forest classification technique was 71.70%(72.10% in the control group and 70.70% in the case group) [24].In 2010, Lin CC in Taiwan established an artificial neural network model and a Logistic regression model to identify metabolic syndrome in 383 patients with schizophrenia, and the results showed that the accuracy was 88.30 and 83.60%, the sensitivity was 93.10 and 86.20%, and the specificity was 86.90 and 83.80%, respectively [25]. In 2015, Worachartcheewa n[26] et al. used the random forest model to establish a prediction model of metabolic syndrome for 5646 adults living in Bangkok, and the accuracy was 98.11%.In 2016, karimi-alavijeh et al. used 2107 participants in the Iranian cohort study to establish the decision-making tree model and support vector machine model, and found that the accuracy was 73.90 and 75.70%, the sensitivity was 75.80 and 77.40%, and the specificity was 72.00 and 74.00 %[27].The established models have local applicability advantages due to the differences in region, population and input variables.

The results of this study showed that the prevalence of MetS in workers of an oil company was 40.67%, higher than the average level of Chinese adults [12–14].At the same time, the prevalence rate of the five diagnostic criteria of metabolic syndrome ranged from high to low, which were: central obesity, abnormal blood pressure, abnormal blood glucose, abnormal triglyceride, and abnormal high-density lipoprotein. The occurrence of this phenomenon was related to the generally good living conditions, dietary habits, irregular life and rest oil workers. According to the importance of predictive variables in the three models established, it was found that the top four variables were age, ALT, BMI and UA, indicating that these four factors played a very important role in the development of metabolic syndrome.In the process of independent variable screening, age, income, BMI, family history of diabetes, salt intake, physical exercise and other factors were the influencing factors of metabolic syndrome, which was consistent with previous results [28, 29]. UA and ALT were found to be risk factors for MetS, and related studies showed that UA increased the risk of MetS by increasing insulin resistance, and increased ALT in the blood might cause fat accumulation in the liver. Through investigation, Mandana Khalili et al. found that patients with MetS had higher hepatic steatosis level, and there was a correlation between the elevation of ALT and MetS [30, 31].Different from the general population, oil workers have been in a special occupational environment for a long time. High temperature environment causes the body’s circulatory system to be in a long-term stress state, resulting in decreased elasticity of blood vessel wall, increased blood viscosity, and increased blood pressure. In addition, studies have shown that high temperature contact can affect insulin hemodynamics, resulting in insulin resistance in the body [32, 33].Harmony between biological rhythm and natural rhythm is the basis of normal physiological activities. Irregular shift work will affect the biological rhythm of human body due to irregular circadian rhythm, resulting in the disturbance of nutrients and related hormones in the body, thus resulting in glucose and lipid metabolism disorder and energy imbalance [34].On the other hand, the workers of night shift work lack of sleep time, and the incidence of unhealthy lifestyle such as smoking, drinking and irregular diet increases greatly, which are the driving forces for the occurrence of metabolic syndrome [35].

In this study, Logistic regression model, random forest model and convolutional neural network model were established to compare their prediction performance. In this study, it was found that the random forest model had higher discriminance and calibration, and was more suitable for the risk prediction of metabolic syndrome of oil workers. As a prediction model for the risk of metabolic syndrome in petroleum workers, the model with higher discrimination is more suitable for the early detection of patients, so as to play a real role in early detection, early diagnosis and early treatment of the disease, namely secondary prevention of the disease. A good clinical disease risk prediction model should not only have good discrimination, but also consider whether it is well calibrated. In this study, Brier Score, O/E ratio, calibration-in-the-large, and Integrated Calibration Index (ICI)were also introduced to evaluate the calibration degree of the model. Among them, the IC I[36] refers to the weighted average of the absolute average difference between the observed probability and the predicted probability, and can be used to quantify the calibration method in the results of dichotomization, so as to evaluate the calibration effect more comprehensively. As an emerging machine learning algorithm in recent years, random forest mode l[37, 38] is a highly flexible classifier containing multiple decision trees. The random forest model solves the shortcoming of the decision tree algorithm, and adopts the random sampling method to enhance the generalization ability. Proposed by Yann Lecun of New York university in 1988, the convolutional neural network model is the first truly successful deep learning method using multi-layer hierarchical network, including input layer, hidden layer (convolutional layer, pooling layer, full connection layer) and output layer, which effectively reduces the number of network parameters and significantly reduces the computational complexity. Previously, convolutional neural network was mainly used for image, language and medical imaging processing. In recent years, it has also been used as a neural network model to predict the risk of various diseases [39–41].However, the prediction effect of CNN for different diseases is uneven, which may be because the model construction needs to be further improved and there is no unified standard yet. At the same time, a certain amount of data is required for model training. Logistic regression model is a traditional statistical modeling method, which is widely used in the field of risk factor screening and disease prediction. It is convenient to use and the meaning of the parameters is clear, but it cannot solve the nonlinear problems and the application conditions are strict. The sample size increases with the increase of input variables, and the predictive power decreases when the data do not meet the requirements [42].

Due to the limitation of research conditions, this study has certain limitations. This paper only developed and internally validated the metabolic syndrome risk prediction model for oil workers, and did not conduct external validation of the model. The choice of model input variables will directly affect the prediction effect of the model, which needs to be further explored.This study was based on a cross-sectional study.Only the prevalence data of metabolic syndrome of oil workers were available, and the causal relationship between the prevalence and predictive factors could not be determined.

## Conclusions

Three risk prediction models (Logistic regression model, random forest model and convolutional neural network model) for the occurrence of metabolic syndrome in petroleum workers were established and compared. The results show that the random forest model has better discriminant degree and calibration degree, and has higher robustness. It shows that the random forest model can predict the risk of metabolic syndrome in oil workers more accurately, and can provide health education for high-risk employees with metabolic syndrome and put forward corresponding prevention strategies, so as to improve the allocation of national medical and health resources and the distribution of health services.

## Supplementary Information

**Additional file 1: Supplementary Table 1.** Coefficient of correlation.

**Additional file 2: Supplementary Table 2.** Results of tolerance and variance inflation factor.

**Additional file 3: Supplementary Figure 1.** The importance of predictive variables in Logistic regression model.

**Additional file 4: Supplementary Figure 2.** The importance of predictive variables in random forest model.

**Additional file 5: Supplementary Figure 3.** The importance of predictive variables in CNN.

**Additional file 6.** I: Logistic regression model code. II: CNN code. III: Random forest model code. IV: Risk score code.

## Data Availability

The data that support the findings of this study are available from [Institute of basic medicine, Chinese academy of medical sciences] but restrictions apply to the availability of these data, which were used under license for the current study, and so are not publicly available. Data are however available from the authors upon reasonable request and with permission of [Institute of basic medicine, Chinese academy of medical sciences].

## Abbreviations

MetS: Metabolic Syndrome
WHO: World Health Organization
NCEP ATP III: National Cholesterol Education Program Adult Treatment group report for the third time
CDS: Chinese Diabetes association
IDF: International Diabetes Federation
AHA: American Heart Association
BMI: Body Mass Index
RBC: Red Blood Cell
MCV: Erythrocyte Mean Corpuscular Volume
BPC: Blood Platelet Count
MPV: Mean Platelet Volume
UA: Uric Acid
ALT: Alanine transaminase
OR: Odds ratio
95%CI: 95% Confident limit
SE: Standard Error
VIF: Variance Inflation Factor
CNN: Convolution Neural Network
AUC: Area Under the Curve
ICI: Integrated Calibration Index
O/E ratio: Observed-expected ratio
