# Supplementary material for: Development and internal validation of risk prediction model of metabolic syndrome in oil workers
Source: BMC Public Health. 2020 Nov 30;20:1828. doi: 10.1186/s12889-020-09921-w (PMC7706262; doi:10.1186/s12889-020-09921-w)
Supplement: Supplementary file 1 — Additional file 1: Supplementary Table 1. Coefficient of correlation. [file 12889_2020_9921_MOESM1_ESM.docx]

Supplementary Table 1. Coefficient of correlation

| Variable name | X_1_ | X_2_ | X_3_ | X_4_ | X_5_ | X_6_ | X_7_ | X_8_ | X_9_ | X_10_ | X_11_ | X_12_ | X_13_ |
| --- | --- | --- | --- | --- | --- | --- | --- | --- | --- | --- | --- | --- | --- |
| X_1_ | 1 |  |  |  |  |  |  |  |  |  |  |  |  |
| X_2_ | 0.062* | 1 |  |  |  |  |  |  |  |  |  |  |  |
| X_3_ | -0.008 | -0.110** | 1 |  |  |  |  |  |  |  |  |  |  |
| X_4_ | 0.068** | 0.022 | 0.014 | 1 |  |  |  |  |  |  |  |  |  |
| X_5_ | -0.021 | -0.015 | 0.141** | -0.008 | 1 |  |  |  |  |  |  |  |  |
| X_6_ | -0.063* | -0.004 | -0.124** | -0.009 | -0.070** | 1 |  |  |  |  |  |  |  |
| X_7_ | -0.147** | -0.001 | 0.010 | -0.010 | 0.065* | 0.288** | 1 |  |  |  |  |  |  |
| X_8_ | -0.019 | -0.016 | -0.043 | -0.034 | -0.027 | -0.034 | -0.045 | 1 |  |  |  |  |  |
| X_9_ | 0.012 | -0.137** | 0.108** | -0.012 | 0.165** | -0.093** | 0.034 | -0.130** | 1 |  |  |  |  |
| X_10_ | 0.018 | 0.310** | 0.081** | 0.054* | 0.004 | -0.052* | 0.011 | 0.039 | -0.012 | 1 |  |  |  |
| X_11_ | 0.028 | -0.044 | 0.091** | 0.028 | 0.000 | -0.047 | 0.005 | 0.040 | 0.028 | 0.047 | 1 |  |  |
| X_12_ | -0.055* | -0.021 | 0.169** | 0.012 | 0.043 | -0.092** | 0.035 | -0.035 | 0.109** | -0.041 | 0.066* | 1 |  |
| X_13_ | 0.084** | 0.015 | 0.168** | 0.058* | 0.026 | -0.110** | -0.042 | -0.078** | 0.049 | 0.006 | 0.090** | 0.226** | 1 |

** P<0.05 ** P<0.01*
