# Supplementary material for: Development and internal validation of risk prediction model of metabolic syndrome in oil workers
Source: BMC Public Health. 2020 Nov 30;20:1828. doi: 10.1186/s12889-020-09921-w (PMC7706262; doi:10.1186/s12889-020-09921-w)
Supplement: Supplementary file 2 — Additional file 2: Supplementary Table 2. Results of tolerance and variance inflation factor. [file 12889_2020_9921_MOESM2_ESM.docx]

Supplementary Table 2. Results of tolerance and variance inflation factor

| Model | Collinearity statistics | |
| --- | --- | --- |
|  | [Tolerance](http://dict.youdao.com/w/tolerance/#keyfrom=E2Ctranslation) | VIF |
| (constant) | - | - |
| Age | 0.966 | 1.036 |
| Per capita monthly household income | 0.881 | 1.135 |
| BMI | 0.897 | 1.115 |
| Family history of diabetes mellitus | 0.985 | 1.015 |
| Salt | 0.952 | 1.051 |
| Dairy intake | 0.844 | 1.185 |
| Carbonated beverage intake | 0.872 | 1.147 |
| Physical exercise | 0.963 | 1.038 |
| Smoking status | 0.922 | 1.085 |
| Shift work situation | 0.897 | 1.115 |
| Occupational heat | 0.975 | 1.026 |
| UA | 0.907 | 1.102 |
| ALT | 0.905 | 1.105 |
