# Supplementary figures and images for: Development and internal validation of risk prediction model of metabolic syndrome in oil workers
Source: BMC Public Health. 2020 Nov 30;20:1828. doi: 10.1186/s12889-020-09921-w (PMC7706262; doi:10.1186/s12889-020-09921-w)

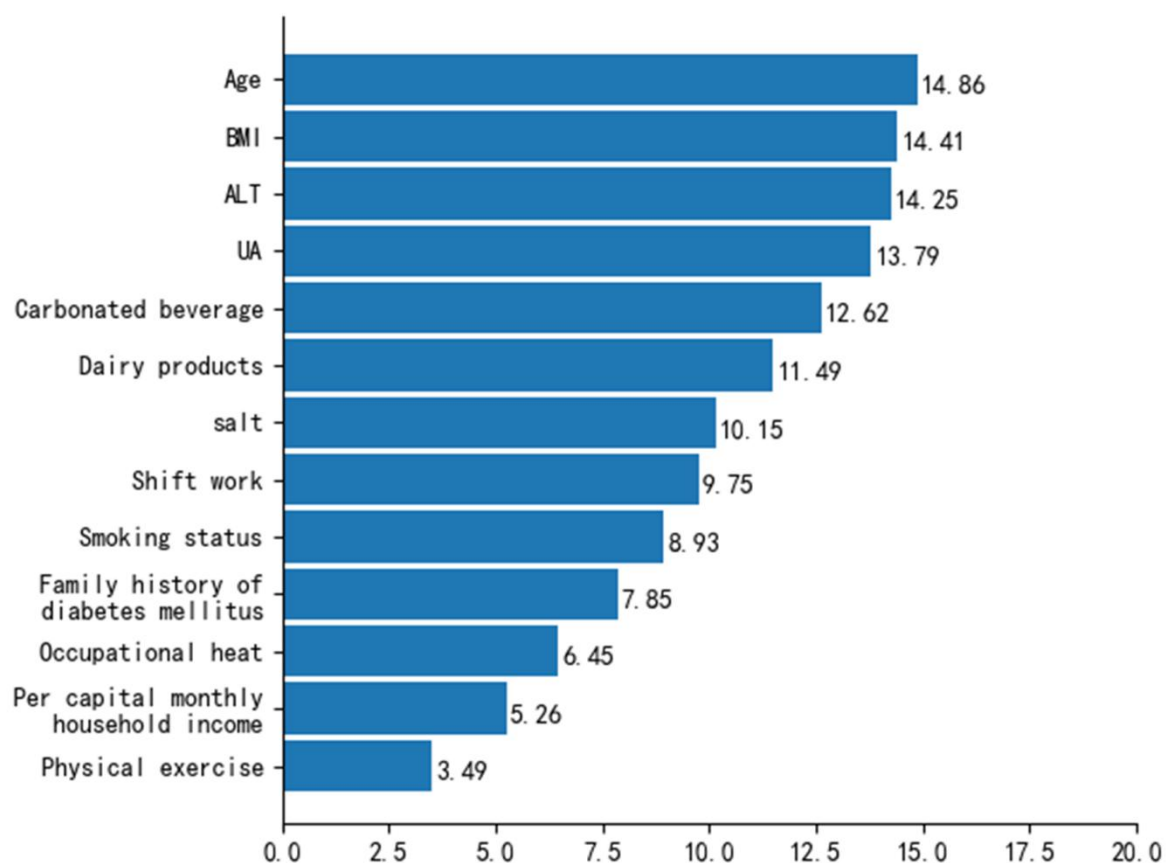

Supplementary Figure 1. The importance of predictive variables in Logistic regression model

Supplement: Supplementary file 3 — Additional file 3: Supplementary Figure 1. The importance of predictive variables in Logistic regression model. [file 12889_2020_9921_MOESM3_ESM.pdf]

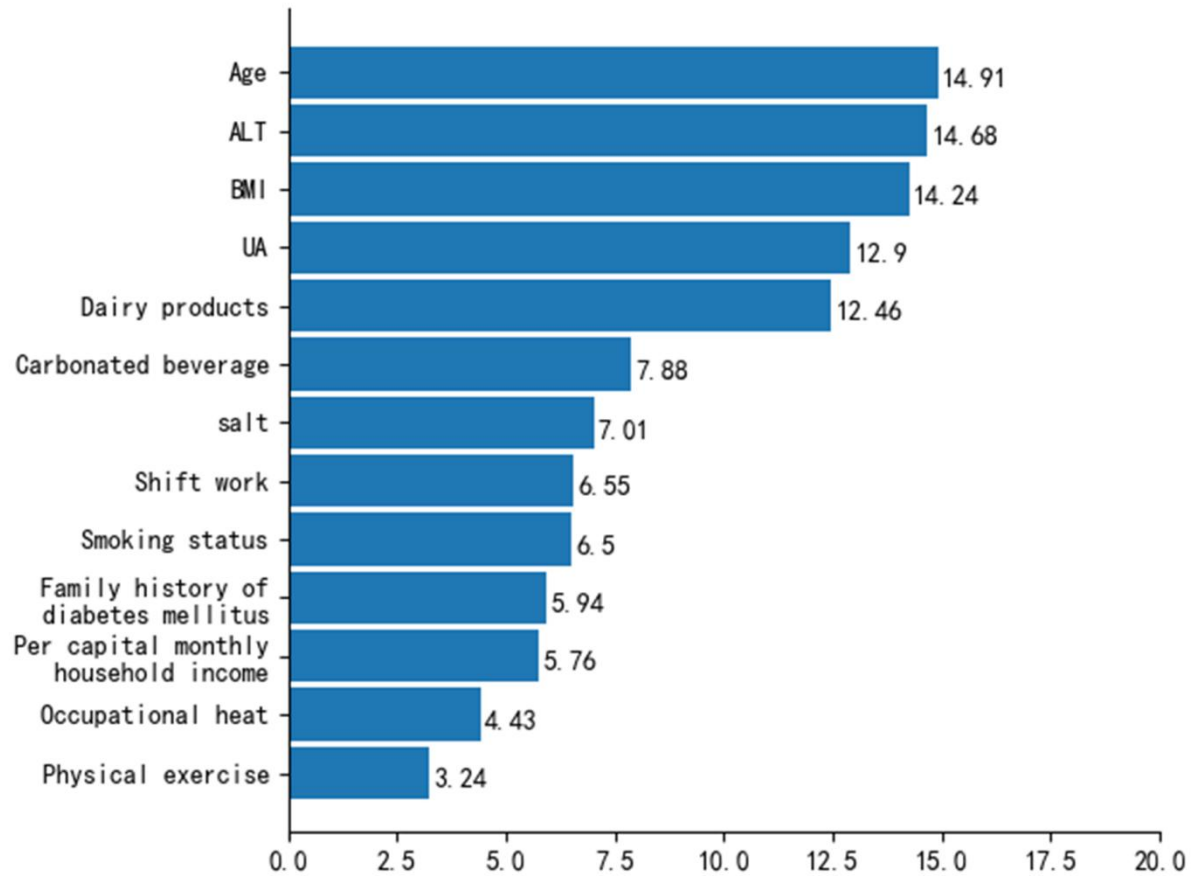

Supplementary Figure 2. The importance of predictive variables in random forest model

Supplement: Supplementary file 4 — Additional file 4: Supplementary Figure 2. The importance of predictive variables in random forest model. [file 12889_2020_9921_MOESM4_ESM.pdf]

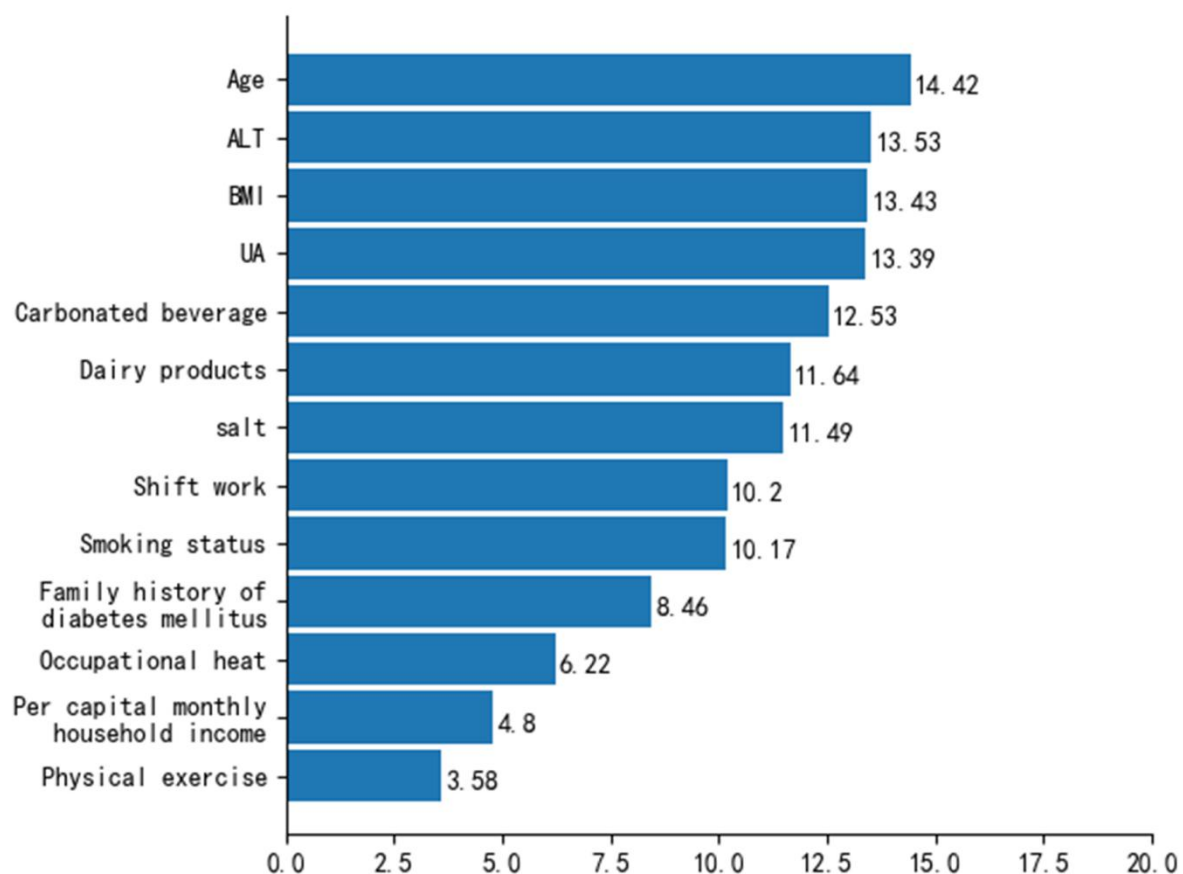

Supplementary Figure 3. The importance of predictive variables in CNN

Supplement: Supplementary file 5 — Additional file 5: Supplementary Figure 3. The importance of predictive variables in CNN. [file 12889_2020_9921_MOESM5_ESM.pdf]
