# Supplementary material for: Development and internal validation of risk prediction model of metabolic syndrome in oil workers
Source: BMC Public Health. 2020 Nov 30;20:1828. doi: 10.1186/s12889-020-09921-w (PMC7706262; doi:10.1186/s12889-020-09921-w)
Supplement: Supplementary file 6 — Additional file 6. I: Logistic regression model code. II: CNN code. III: Random forest model code. IV: Risk score code. [file 12889_2020_9921_MOESM6_ESM.docx]

**Ⅰ.Logistic regression model**

#1.Split training set and test set

from sklearn.cross_validation import train_test_split

exam_X=exam_X.values.reshape(-1,1)

exam_y=exam_y.values.reshape(-1,1)

train_X,test_X,train_y,test_y=train_test_split(exam_X,exam_y,train_size=0.8)

#2.Import model

from sklearn.linear_model import LogisticRegression

modelLR=LogisticRegression()

#3.Training model

modelLR.fit(train_X,train_y)

#4.Model evaluation

modelLR.score(test_X,test_y)

#5.Let's figure out the regression function y=a+bx, and then let's plug it in to the logical functionpred_y=1/(1+np.exp(-y))

b=modelLR.coef_

a=modelLR.intercept_

print('The regression function corresponding to this model is:1/(1+exp-(%f+%f*x))'%(a,b))

from sklearn.metrics import confusion_matrix

#6. Numerical treatment

pred_y=1/(1+np.exp(-(a+b*test_X)))

pred_y=pd.DataFrame(pred_y)

pred_y=round(pred_y,0).astype(int)

#7. Confusion matrix

confusion_matrix(test_y.astype(str),pred_y.astype(str))

#8.Draw the model ROC curve

from sklearn.metrics import roc_curve, auc

# Compute ROC curve and ROC area for each class

fpr,tpr,threshold = roc_curve(test_y, pred_y)

roc_auc = auc(fpr,tpr)

plt.figure()

lw = 2

plt.figure(figsize=(10,10))

plt.plot(fpr, tpr, color='r',

lw=lw, label='ROC curve (area = %0.2f)' % roc_auc)

plt.plot([0, 1], [0, 1], color='navy', lw=lw, linestyle='--')

plt.xlim([0.0, 1.0])

plt.ylim([0.0, 1.0])

plt.xlabel('False Positive Rate')

plt.ylabel('True Positive Rate')

plt.title('Receiver operating characteristic example')

plt.legend(loc="lower right")

plt.show()

**Ⅱ.CNN**

# 2. Import libraries and modules

from keras.models import Sequential

from keras.layers import Conv2D, MaxPool2D

from keras.layers import Dense, Flatten

from keras.utils import to_categorical

# 3. Data loading

from keras.datasets import mnist

(x_train, y_train), (x_test, y_test) = mnist.load_data()

# 4. Data preprocessing

img_x, img_y = 28, 28

x_train = x_train.reshape(x_train.shape[0], img_x, img_y, 1)

x_test = x_test.reshape(x_test.shape[0], img_x, img_y, 1)

x_train = x_train.astype('float32')

x_test = x_test.astype('float32')

x_train /= 255

x_test /= 255

y_train = to_categorical(y_train, 10)

y_test = to_categorical(y_test, 10)

# 5. Define the model structure

model = Sequential()

model.add(Conv2D(32, kernel_size=(5,5), activation='relu', input_shape=(img_x, img_y, 1)))

model.add(MaxPool2D(pool_size=(2,2), strides=(2,2)))

model.add(Conv2D(64, kernel_size=(5,5), activation='relu'))

model.add(MaxPool2D(pool_size=(2,2), strides=(2,2)))

model.add(Flatten())

model.add(Dense(1000, activation='relu'))

model.add(Dense(10, activation='softmax'))

# 6. Compiling

model.compile(optimizer='adam',

loss='categorical_crossentropy',

metrics=['accuracy'])

# 7. Training

model.fit(x_train, y_train, batch_size=128, epochs=10)

# 8. Model evaluation

score = model.evaluate(x_test, y_test)

print('acc', score[1])

**Ⅲ.Random forest model**

import pandas as pd

from sklearn.model_selection import KFold

import numpy as np

df = pd.read_excel('try.xlsx')

# print(df.loc[0])

y = df['MS ill']

num = df['Number']

df.drop(['Number','MS ill'], axis=1, inplace=True)

# print(df.loc[0])

# from sklearn.model_selection import train_test_split

# x_train,x_test,y_train,y_test = train_test_split(df.values,y,test_size=0.3,random_state=17)

def forest(x_train,y_train,x_test,y_test,y_true,count,y_trues,y_propres,cons):

# print('df.values = ',df.values)

# print("count = ",count)

import numpy as np

from sklearn.ensemble import RandomForestClassifier

from sklearn.model_selection import GridSearchCV

from sklearn.metrics import accuracy_score,confusion_matrix,brier_score_loss

# print(np.shape(x_train))

forest = RandomForestClassifier(n_estimators=100,n_jobs=-1,random_state=17)

forest_params = {'max_depth': range(8, 10),

'max_features': range(5, 7)}

#

forest_grid = GridSearchCV(forest, forest_params,

cv=10, n_jobs=-1, verbose=True)

#

forest_grid.fit(x_train, y_train)

forest_grid.best_params_, forest_grid.best_score_

#

b = accuracy_score(y_test, forest_grid.predict(x_test))

print("score:",1/b)

y_pre = forest_grid.predict(x_test)

con=np.array(confusion_matrix(y_true,y_pre))

# print(con)

# print(type(con))

cons.append(con)

# print('y_pre = ',y_pre)

# print('y = ',y)

from sklearn.metrics import roc_curve, auc, accuracy_score

from sklearn.calibration import calibration_curve

import matplotlib.pyplot as plt

y_true = np.array(y_true)

y_pre = np.array(y_pre)

bp = np.power(y_true-y_pre,2)

print(bp)

print("The weighted average ICI is：")

print(np.sqrt(np.sum(bp)))

y_propre = np.array(forest_grid.predict_proba(x_test))

plt.plot(calibration_curve(y_true,y_propre[:,1]))

# print(y_propre)

# print(y_propre[:,1])

# print(y_propre)

# print("len_y_true = ",len(y_true))

# print("len_y_propre = ",len(y_propre))

# print(brier_score_loss(y_true,y_propre[:,1]))

fpr,tpr,thresholds = roc_curve(y_true,y_propre[:,1],pos_label=1)

# print('y_true = ',len(y_true))

# print('y_pre = ',len(y_pre))

# print('fpr = ',len(fpr))

# print('tpr = ',len(tpr))

# print("fpr = ",fpr)

# print("tpr = ",tpr)

# print("len_fpr = ", len(fpr))

# print("len_tpr = ", len(tpr))

# print("type_fpr = ",type(fpr))

# print("type_tpr = ",type(tpr))

# print(type(tpr))

y_trues.append(y_true)

print(type(y_true))

print(type(y_pre))

print(len(y_true))

print(len(y_pre))

y_propres.append(y_propre)

roc_auc = auc(fpr,tpr)

plt.plot(fpr, tpr, 'r--', label='ROC (area = {0:.2f})'.format(roc_auc), lw=2)

plt.plot([0, 1], [0, 1], color='navy', lw=2, linestyle='--')

plt.xlim([-0.05, 1.05])

plt.ylim([-0.05, 1.05])

plt.xlabel('True Positive Rate')

plt.ylabel('False Positive Rate')

plt.title('ROC Curve')

plt.legend(loc="lower right")

plt.show()

# print(np.array(tprs))

# print(np.array(fprs))

# print(type(np.array(tprs)))

# print(np.array(tprs).shape)

if count == 10:

# print("mean：")

y_trues = np.array(y_trues)

y_propres = np.array(y_propres)

print("Possibility for： 0 1")

print(y_propres)

cons = np.array(cons)

# print(cons)

# print("The confusion matrix is：")

sum_con=np.sum(cons,axis=0)

# print(sum_con)

ave_y_true = y_trues.mean(axis=0)

ave_y_propre = y_propres.mean(axis=0)

# print("ave_y_true**type =",np.shape(ave_y_true))

# print("ave_y_true**type =", np.shape(ave_y_propre))

# print("ave_y_true**length = ",ave_y_true))

# print(ave_y_true)

# print("ave_fpr = ",ave_fpr)

# print("ave_tpr = ",ave_tpr)

# print("len_ave_fpr = ", len(ave_fpr))

# print("len_ave_tpr = ", len(ave_tpr))

# print("type_ave_fpr = ", type(ave_fpr))

# print("type_ave_tpr = ", type(ave_tpr))

ave_fpr,ave_tpr,ave_thresholds = roc_curve(ave_y_true,ave_y_propre[:,1],pos_label=1)

ave_roc_auc = auc(ave_fpr,ave_tpr)

plt.plot(ave_fpr, ave_tpr, 'r--', label='Are_ROC (area = {0:.2f})'.format(ave_roc_auc), lw=2)

plt.plot([0, 1], [0, 1], color='navy', lw=2, linestyle='--')

plt.xlim([-0.05, 1.05])

plt.ylim([-0.05, 1.05])

plt.xlabel('Ave_False Positive Rate')

plt.ylabel('Ave_True Positive Rate')

plt.title('Ave_ROC Curve')

plt.legend(loc="lower right")

plt.show()

kf = KFold(n_splits=10,shuffle=True)

count = 0

cons=[]

for i, j in kf.split(df.values,y):

x_train,y_train = df.values[i],y[i]

x_test,y_test = df.values[j],y[j]

y_true = list(y_test)

y_trues = []

y_propres = []

count+=1

if count== 10:

break

forest(x_train,y_train,x_test,y_test,y_true,count,y_trues,y_propres,cons)

a = np.array([[766, 105], [152, 445]])

b = np.array([[789, 82], [35, 562]])

c = np.array([[832, 39], [20, 577]])

print("brier_score of Logistic")

# print("The confounding matrix of logistic regression：")

print(a)

print("The confounding matrix of CNN：")

print(b)

print("The confounding matrix of random forest：")

print(c)

**Ⅳ Risk score**

import numpy as np

import pandas as pd

data = pd.read_csv('D:\desktop\give me some credit\give+me+some+cridits\cs-training.csv')

data = data.iloc[:,1:]

data.head()

data.shape

data.describe()

data.info()

from sklearn.ensemble import RandomForestRegressor

def add_missing(df):

process_df = df.ix[:,[5,0,1,2,3,4,6,7,8,9]]#Select only some columns,

#It is divided into two parts: known eigenvalue and eigenvalue of position

know = process_df[process_df.MonthlyIncome.notnull()].as_matrix()

unknow = process_df[process_df.MonthlyIncome.isnull()].as_matrix()

Y = know[:,0]

X = know[:,1:]

rfr = RandomForestRegressor(random_state = 0, n_estimators=200, max_depth=3, n_jobs = -1)

rfr.fit(X,Y)

predicted = rfr.predict(unknow[:,1:])

df.loc[df['MonthlyIncome'].isnull(), 'MonthlyIncome'] = predicted

return df

data = add_missing(data)

Handling outliers

import matplotlib.pyplot as plt

%matplotlib inline

data_box = data.iloc[:,[2,3,7,9]]

data_box.boxplot()

def self_bin(Y,X,cat):

good=Y.sum()

bad=Y.count()-good

d1=pd.DataFrame({'X':X,'Y':Y,'Bucket':pd.cut(X,cat)})

d2=d1.groupby(['Bucket'])

d3=pd.DataFrame(d2['X'].min(),columns=['min'])

d3['min']=d2['X'].min()

d3['max']=d2['X'].max()

d3['sum']=d2['Y'].sum()

d3['total']=d2['Y'].count()

d3['rate']=d2['Y'].mean()

d3['goodattribute']=d3['sum']/good

d3['badattribute']=(d3['total']-d3['sum'])/bad

d3['woe']=np.log(d3['goodattribute']/d3['badattribute'])

iv=((d3['goodattribute']-d3['badattribute'])*d3['woe']).sum()

d4=d3.sort_index(by='min')

print(d4)

print('-'*40)

woe=list(d3['woe'].values)

return d4,iv,woe

ninf=float('-inf')

pinf=float('inf')

cutx3=[ninf,0,1,3,5,pinf]

cutx6 = [ninf, 1, 2, 3, 5, pinf]

cutx7 = [ninf, 0, 1, 3, 5, pinf]

cutx8 = [ninf, 0,1,2, 3, pinf]

cutx9 = [ninf, 0, 1, 3, pinf]

cutx10 = [ninf, 0, 1, 2, 3, 5, pinf]

dfx3,ivx3,woex3=self_bin(train['SeriousDlqin2yrs'],train['NumberOfTime30-59DaysPastDueNotWorse'],cutx3)

dfx6, ivx6 ,woex6= self_bin(train['SeriousDlqin2yrs'], train['NumberOfOpenCreditLinesAndLoans'], cutx6)

dfx7, ivx7,woex7 = self_bin(train['SeriousDlqin2yrs'], train['NumberOfTimes90DaysLate'], cutx7)

dfx8, ivx8,woex8 = self_bin(train['SeriousDlqin2yrs'], train['NumberRealEstateLoansOrLines'], cutx8)

dfx9, ivx9,woex9 = self_bin(train['SeriousDlqin2yrs'], train['NumberOfTime60-89DaysPastDueNotWorse'], cutx9)

dfx10, ivx10,woex10 = self_bin(train['SeriousDlqin2yrs'], train['NumberOfDependents'], cutx10)

import matplotlib.pyplot as plt

%matplotlib inline

ivall = pd.Series([ivx1,ivx2,ivx3,ivx4,ivx5,ivx6,ivx7,ivx8,ivx9,ivx10],index = ['x1','x2','x3','x4','x5','x6','x7','x8','x9','x10'])

fig = plt.figure()

ax1 = fig.add_subplot(111)

ivall.plot.bar()

plt.show
